# Optically pumped magnetometers: From quantum origins to multi-channel magnetoencephalography

**DOI:** 10.1016/j.neuroimage.2019.05.063

**Published:** 2019-10-01

**Authors:** Tim M. Tierney, Niall Holmes, Stephanie Mellor, José David López, Gillian Roberts, Ryan M. Hill, Elena Boto, James Leggett, Vishal Shah, Matthew J. Brookes, Richard Bowtell, Gareth R. Barnes

**Affiliations:** a Wellcome Centre for Human Neuroimaging, UCL Institute of Neurology, London, WC1N 3AR, UK; b Sir Peter Mansfield Imaging Centre, School of Physics and Astronomy, University of Nottingham, University Park, Nottingham, NG7 2RD, UK; c Engineering Faculty, Universidad de Antioquia UDEA, Calle 70 No 52-21, Medellín, Colombia; d QuSpin Inc., 331 South 104th Street, Suite 130, Louisville, CO, 80027, USA

## Abstract

Optically Pumped Magnetometers (OPMs) have emerged as a viable and wearable alternative to cryogenic, superconducting MEG systems. This new generation of sensors has the advantage of not requiring cryogenic cooling and as a result can be flexibly placed on any part of the body. The purpose of this review is to provide a neuroscience audience with the theoretical background needed to understand the physical basis for the signal observed by OPMs. Those already familiar with the physics of MRI and NMR should note that OPMs share much of the same theory as the operation of OPMs rely on magnetic resonance. This review establishes the physical basis for the signal equation for OPMs. We re-derive the equations defining the bounds on OPM performance and highlight the important trade-offs between quantities such as bandwidth, sensor size and sensitivity. These equations lead to a direct upper bound on the gain change due to cross-talk for a multi-channel OPM system.

## Highlights

• We review the theoretical basis of OPMs.
• We re-derive the signal equations for OPMs.
• We highlight the important trade-offs in sensor design.
• We discuss the practical implementation of this technology.

## 1 Introduction

Optically Pumped Magnetometers (OPMs) are capable of measuring very weak magnetic fields (femtotesla sensitivity) without the need for cryogenic cooling (Shah and Wakai, 2013). This ability means that OPMs can be used to develop a new generation of more flexible and highly sensitive Magnetoencephalography (MEG) systems (Boto et al., 2016a, Boto et al., 2016b). To date, MEG has typically been implemented using Superconducting QUantum Interference Devices (SQUIDs). This technology has been tremendously successful and has established MEG as an essential neuroscience tool in many laboratories (Baillet, 2017).

OPMs operate on very different physical principles to SQUIDs. Principally, they do not require cryogenic cooling and can be placed within millimetres of the subject's scalp. This simple advantage of bringing the sensors closer to the subject's brain offers a three-to five-fold improvement in sensitivity (as well as consistency across different headshapes and sizes) to cortical sources compared to traditional SQUID based MEG (Boto et al., 2016a; Iivanainen, Stenroos and Parkkonen, 2017a; Sander et al., 2012a). Furthermore, the wearability and motion robustness of an OPM-MEG system (Boto et al., 2018; Holmes et al., 2018) means that novel neuroscientific questions that involve subject movement can be asked and answered with OPMs. There should also be an additional clinical benefit especially in traditionally less-compliant patient populations (Yerys et al., 2009). For example, pre-surgical planning based on spike-localization and localization of eloquent cortex in paediatric epilepsy (Bagic et al., 2017). In this case we know that young children stand to benefit most from early epilepsy surgery as they have improved prospects to return to a normal developmental trajectory the younger the surgery takes place (Cross et al., 2006; Simon and Rosenfeld, 2013). We also anticipate benefits at the other end of the age spectrum, with (for example) the ability to robustly scan patients suffering from stroke or movement disorders who might have difficulty complying with the constraints of conventional static systems.

Advantages aside, the purpose of this work is to provide the theoretical background to understand OPMs ranging from the quantum mechanics which underpins their operation to their modern day usage in wearable, MEG systems. The interested scientist will quickly note that much of the theory necessary to understand OPMs is shared with Nuclear Magnetic Resonance (NMR) and Magnetic Resonance Imaging (MRI). The article continues with a brief history of OPM development and a non-technical summary of the operation of these sensors. We then go on to describe the fundamentals of optical pumping which in turn leads on to a formal description of OPM operation. These quantitative accounts give rise to an insight into the key physical parameters governing system performance and hence future research directions.

## 2 History and non-technical overview

### 2.1 A brief history

Both MRI and OPMs rely on the manipulation of a quantum property know as spin (a property that underlies a particle's magnetic moment and therefore its response to a magnetic field). The studies characterising the nuclear and electron spin were first described in the 1920s and 1930s (Rabi et al., 1938; Stern and Gerlach, 1922; Uhlenbeck and Goudsmit, 1926) and this property forms the basis for NMR, MRI and OPMs. While optically pumped magnetometry and MRI are both fundamentally based on the manipulation of spins they differ in the way that the spin systems are manipulated. In NMR and MRI a large magnetic field is generally used to affect the nuclear spin system (Bloch et al., 1946) while with OPMs optical pumping is used to manipulate the atomic (i.e. both nuclear and electron) spin (Kastler, 1951).

Optical pumping refers to the use of a light source such as a laser or discharge lamp (Bell and Bloom, 1957; Groeger et al., 2005) to cause absorption or emission of energy by a sample (usually a vapour formed of one of the alkali metals due to their simple atomic structure, low melting point and ease of pumping with readily available lasers) at a precisely defined frequency, changing the sample's quantum state. As the frequency (inverse of period) at which these absorptions and emissions occurs is known with great precision optical pumping of alkali metals can be used to help define the SI unit of time (Bender et al., 1958; Markowitz et al., 1958). This approach gave birth to the atomic clock. The interested reader is directed to a review of the roles atomic transitions in alkali vapours (fundamental components of OPMs) have had in the definition of time (Lombardi et al., 2007).

In the late 1950s and early 1960s it was shown that optical pumping could be used for inducing a magnetically sensitive state in an atomic system and therefore allow for the measurement of weak magnetic fields (Bell and Bloom, 1957; Bloom, 1962). By the beginning of the 1970s OPMs were achieving sensitivities in the range of 30fT/√Hz by applying modulation fields to improve SNR and operating the sensors in a magnetically shielded environment (Cohen-Tannoudji et al., 1970; Dupont-Roc et al., 1969; Kastler, 1973; Slocum et al., 1973). Soon after it was shown that by increasing the density of the alkali vapour the sensors could maintain a high degree of magnetic sensitivity (Happer and Tang, 1973) which allowed for miniaturisation of the vapour containers and the sensors themselves.

Nearly thirty years later, the magnetic sensitivity has been improved to levels comparable with SQUID systems (Budker et al., 2000). This has been made possible by combing the sensitivity gains from working at zero field with the additional sensitivity offered by working with high density vapours. This regime is often referred to as the spin exchange relaxation free(SERF) regime (Allred et al., 2002; Kominis et al., 2003). Conceptually, this regime minimises the effect colliding particles have on the magnetically sensitive state of the vapour by increasing the rate of collisions (by heating the vapour). This seemingly paradoxical effect will be explored further in later sections.

These improvements in sensitivity, coupled with advances in microfabrication have allowed for the exciting development of compact OPMs that can be utilised for neuroscience applications (Knappe et al., 2016; Osborne et al., 2018; Shah and Wakai, 2013; Sheng et al., 2017a). Coupled with advances in sensors, additional neuroscientific motivation for OPMs came from simulation studies examining issues such as improvements in SNR, spatial resolution and the requirements necessary for accurate source reconstruction (Boto et al., 2016a; Iivanainen et al., 2017a; Zetter et al., 2017).

To date, relatively few empirical OPM-MEG studies exist. A common theme is that these studies have tended to focus on primary sensory systems, generally because to date only limited sensor coverage has been possible. For example, OPMs have been used to study auditory evoked fields (Borna et al., 2017b; C. N. Johnson, Schwindt and Weisend, 2013; C. Johnson, Schwindt and Weisend, 2010; Kim et al., 2014; Labyt et al., 2018; Osborne et al., 2018; Sander et al., 2012a; Shah and Wakai, 2013; J. Sheng et al., 2017b; Xia et al., 2006), somatosensory evoked responses (Borna et al., 2017a; Boto et al., 2016b; C. Johnson et al., 2010; Kamada et al., 2015; Sander et al., 2012b), visual evoked fields (Labyt et al., 2018) and retinotopy (Holmes et al., 2018). Outside of sensory cortex, OPMs have been used to study the motor system while the subject is free to move (Boto et al., 2018) and (in one example of a distributed array) to localise and lateralise language function in the human brain (Tierney et al., 2018). Animal studies have also been performed demonstrating the feasibility for OPMs to record spiking activity in rodent models of epilepsy (Alem et al., 2014).

Over the course of this review we will discuss the technical aspects of these sensors that has allowed them to transition from a method for probing atomic structure to an exciting new tool for neuroscience.

### 2.2 In a nutshell

Here we provide a brief and non-technical description of OPM function before the more detailed explanation which follows. In the simplest of setups an OPM contains three (but there are many variations on this) crucial components: A light source (laser or discharge lamp), a high pressure vapour, and a detection system.

For this simple case the light source may be laser and the detection system may be a photodiode. The laser emits electromagnetic waves that establish a magnetically sensitive state in the vapour by transferring polarised light to the vapour (details expanded upon in later sections). The development of this sensitive state is referred to as *optical pumping*. Importantly, once this optical pumped state is established the light source no longer transfers polarised light to the vapour. Instead the polarised light passes through the vapour and is detected at the photodiode as a change in voltage. However, this optically pumped state is disrupted by the presence of an ambient magnetic field and can only be re-established by the laser once again transferring polarised light to the vapour. As such the output of the photodiode, measured as a voltage, varies as a function of the magnetic field.

In more complicated setups two lasers may be used (Colombo et al., 2016), one for inducing the magnetically sensitive state (usually called the pump beam) and one for measuring the changes in the magnetic field (usually called the probe beam). The pump beam can be circularly polarised, but this depends on the atom being pumped (Labyt et al., 2018). What is important is that both the frequency and polarity of the laser are selected to cause a pumping effect in the high pressure vapour. The probe beam is typically linearly polarised but its frequency is carefully chosen so as not to cause a pumping effect. In the presence of a magnetic field the polarisation of the probe beam rotates (by Faraday rotation) in a manner which is proportional to the state of the vapour (as the state of the vapour is a function of the magnetic field). This change in probe beam polarisation can be measured with a polarimeter. Regardless of whether one or two lasers are used, both situations result in signal that is proportional to the magnetically sensitive state of the vapour (Colombo et al., 2016; Seltzer and Romalis, 2009; Shah and Romalis, 2009).

**Fig. 1:**
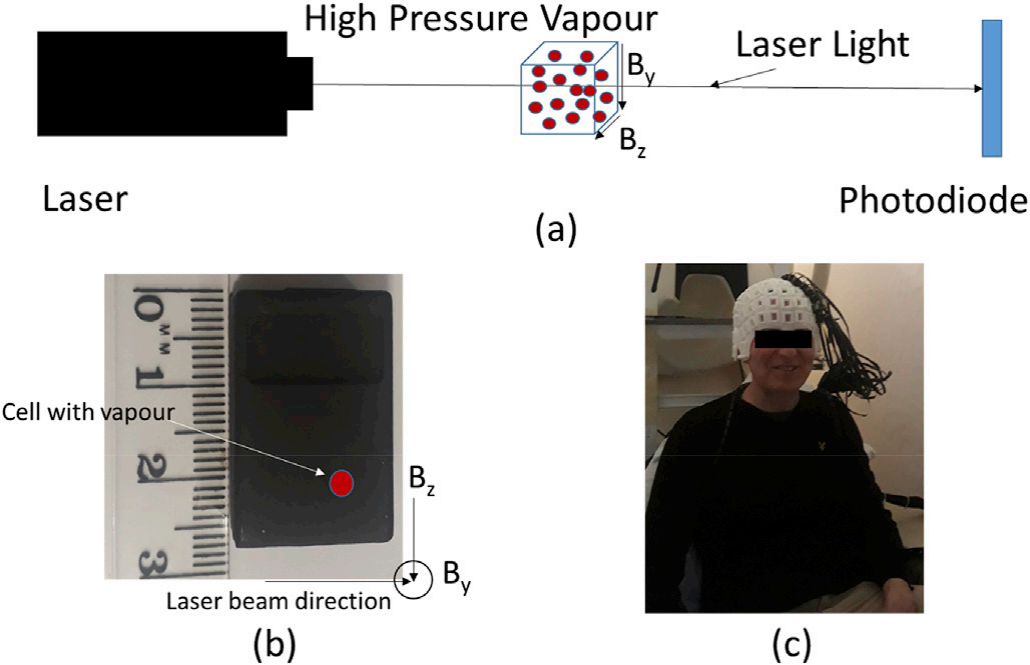
OPM and schematic. In (a) an internal schematic of a general OPM is described. A Laser light is shone through a glass cell containing a vapour under high pressure. The amount of light detected at the photodiode is a function of the ambient magnetic fields perpendicular to the axis of the laser beam (B_z_ and B_y_). In (b) a Gen-2 Quspin OPM can be seen with the directions of the measured magnetic fields, laser and position of vapour cell. In (c) an OPM array of 17 sensors inserted into a wearable scanner-cast is displayed.

There are many other OPM designs, but we will restrict the discussion in this paper to the simple case of the single laser (Fig. 1) as it shares much in common with the pump, probe setup, and many of these other methods have yet to be utilised in the context of MEG.

## 3 Quantum basis of optical pumping

Optical Pumping is a method by which a light source transfers *angular momentum* to a sample in such a way that nearly all atoms in the sample occupy the same *energy level*. The key concepts of *angular momentum* and *energy levels* will be explored further in the following sections.

### 3.1 Angular momentum

In this context angular momentum refers to the quantum mechanical phenomenon that shares the same SI units of $kg\;m^{2}s^{-1}\;$with classical, Newtonian angular momentum. By understanding the total angular momentum of the atom ($\vec{F}$) in a weak magnetic field we can understand how optical pumping can occur. The total angular momentum of an atom ($\vec{F}$), in a weak magnetic field, is the sum of the angular momentum of the nucleus ($\vec{I}$) and electron ($\vec{J}$),

(1) $$\begin{array}{c} \vec{F}=\vec{J}+\vec{I}\; \end{array}$$

Note that throughout this paper, vectoral quantities are distinguished from scalar quantities by notation (e.g.,$\;\vec{F}$ indicates a vectoral quantity whereas F indicates a scalar quantity). The electron angular momentum ($\vec{J}$) is further composed of an orbital angular momentum ($\vec{L}$) and a spin angular momentum ($\vec{S}$),

(2) $$\begin{array}{c} \vec{J}=\vec{L}+\vec{S}\; \end{array}$$

At this point it should be noted that the nuclear angular momentum and spin angular momentum of the electron are not a result of motion, rather this form of angular momentum is “intrinsic” to the atom. Fortunately, one can pragmatically interpret these forms of quantum angular momentum in terms of their observable effects on the real world. Most notably, the total angular momentum $\left(\vec{F}\right)$ is associated with the total magnetic moment of the atom ($\vec{\mu_{F}}$),

(3) $$\begin{array}{c} \vec{\mu_{F}}=\;\gamma \vec{F} \end{array}$$

where γ is the gyromagnetic ratio of the atom (a constant). For ^87^Rb, the element used in many OPMs, γ is a constant with a value of approximately 7 Hz nT^−1^ (Benumof, 1965). In the presence of a magnetic field$\left(\vec{B}\right)$ this magnetic moment is associated with a potential energy(E)

(4) $$\begin{array}{c} E=\;-\vec{\mu_{F}}\cdot \vec{B} \end{array}$$

These relations (eqs(3), (4))) highlight the fundamental relationship between energy, angular momentum, magnetic moment and magnetic field. The take home message from this section is that if for any reason the angular momentum of the atom ($\vec{F}$) changes, this will also change the atom's magnetic moment($\vec{\mu_{F}}$) and potential energy (E). So, therefore, if the laser transfers angular momentum to the atom, the potential energy of the atom will change. This change in energy is an example of an energy level transition. In the next section we will discuss under which particular conditions laser energy can be transferred to the atom to cause these transitions. An interesting property of alkali metals used in OPMs (rubidium, caesium) is that their behaviour is largely determined by the single electron that is furthest from the nucleus. Thus, when we discuss changing *energy levels* it is this electron that is changing state.

### 3.2 Fine structure splitting and the D1/D2 transitions

This outermost electron can exist in a number of discrete states. Transition between these states requires absorption or emission of energy. Fine structure splitting is the name given to the energy level difference arising from the interaction of the electron's orbital angular momentum ($\vec{L}$) and its spin angular momentum ($\vec{S}$). The energy levels defined by this momenta can be described by the introduction of dimensionless quantum numbers (S&L, which can take values of ½ (S) and 0, 1, 2, 3, 4 (L) respectively for ^87^Rb). These numbers relate to the magnitude of the momenta in the following way, $\hbar \;\left(1.054571800\times 10^{-34}\right)$ being the reduced Planck's constant.

(5) $$\begin{array}{c} \left|\vec{S}\right|=\sqrt{S\left(S+1\right)}\hbar \;, \end{array}$$

(6) $$\begin{array}{c} \left|\vec{L}\right|=\sqrt{L\left(L+1\right)}\hbar \; \end{array}$$

**Fig. 2:**
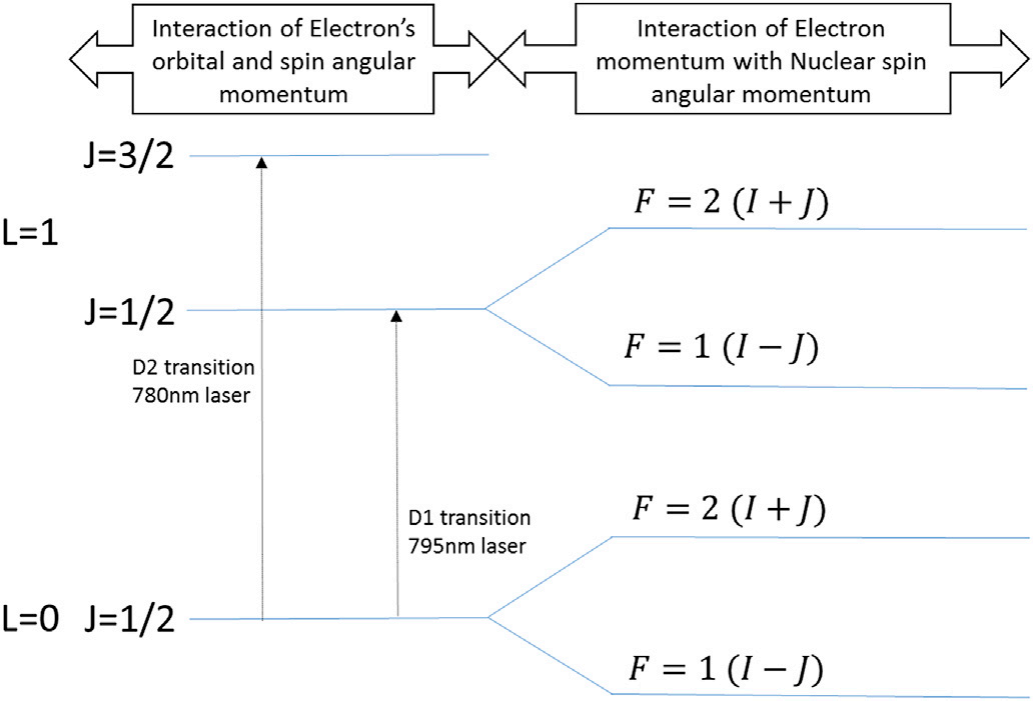
Energy level diagram displaying fine and hyperfine structure splitting of ^87^Rb. The fine structure splitting (left of panel) is a result of the interaction between the electron's orbital and spin angular momentum. The dimensionless number *J* defines the magnitude of the total angular momentum of the electron ($\left|\vec{J}\right|$) in a similar way to which S defines the magnitude of the spin angular momentum of the electron. The hyperfine structure splitting is a result of the interaction between the total angular momentum of the electron (*J*) and the spin angular momentum of the nucleus (*I*), resulting in possible values for total angular electron momentum (*F*) of 2 and 1.

The range of distinct energy levels due to the interaction of the spin angular momentum of the electron ($\vec{S}$) and the orbital angular momentum ($\vec{L}$) are found by taking integer steps in the following range:

(7) $$\begin{array}{c} \left|L-S\right|\leq J\leq L+S\;, \end{array}$$

where J is a dimensionless quantum number which characterises the total angular momentum of the electron. The number of valid values for J define the number of states associated with fine structure splitting. In its ground state, the orbital angular momentum (L) of ^87^Rb is zero. Its spin quantum number (S) is ½. Therefore, in the ground state there is only one possible value of J that satisfies the above equation (J=½). For the first excited state (L=1), there are two possible values of J ($J=\frac{1}{2}$ and $\;J=\frac{3}{2}$) that satisfy the above relation. Transitions to these two states from L = 0 are known, by convention, as the D1 and D2 transitions respectively (Fig. 2). This means that when we excite the atom from L=0to L=1 (by application of laser light), there are two possible J states the atom can exist in. We can selectively choose which energy level the atom occupies by choosing the appropriate frequency of laser light as the energy (E) each photon carries is equal to its frequency(v) multiplied by Planck's constant (h),

(8) $$\begin{array}{c} E=hv\; \end{array}$$

In ^87^Rb, by using a laser with a 795 nm wavelength one can select only D1 transitions (i.e. to the $L=1,\;\;J=\frac{1}{2}$ state).

### 3.3 Hyperfine structure splitting

The *fine structure* (interaction between the electron's orbital ($\vec{L}$) and spin ($\vec{S}$) angular momentum) of the atom is further split by interaction of the electron's total angular momentum ($\vec{J}$) and the nucleus' angular momentum ($\vec{I}$). The dimensionless quantum number, F(defines possible hyperfine states) which characterises this energy level splitting (similar to eq (7)) is simply,

(9) $$\begin{array}{c} \left|I-J\right|\leq F\leq I+J \end{array}$$

Due to the relative contribution of protons and neutrons in the nucleus, the dimensionless quantum number I has a value of 3/2 for ^87^Rb (Levitt, 2000). As noted in the previous section, by only causing a D1 transition J will always have a value of ½. Therefore F  = 1 or 2. This is graphically represented in the energy level diagram in Fig. 2.

### 3.4 Zeeman splitting and optical pumping

**Fig. 3:**
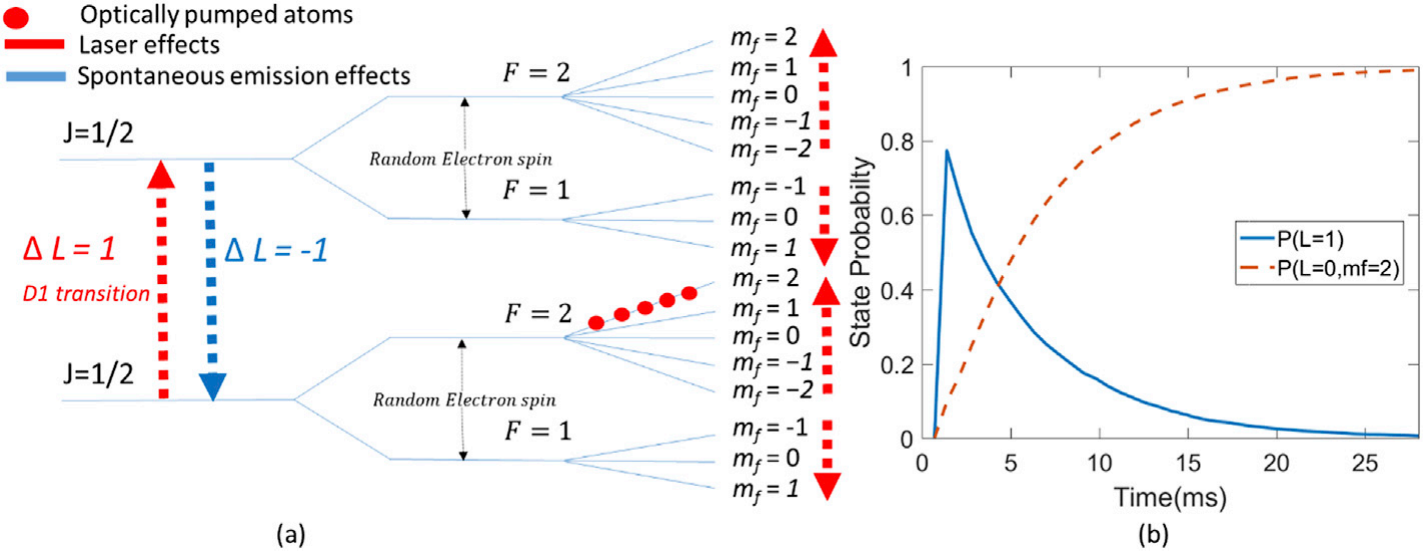
Optical pumping of ^87^Rb. In (a) the laser will always provide an increment in $m_{f}$ and, if possible, a D1 transition (from *L* = 0 to *L* = 1). This transition however will only be possible if $\;m_{f}$ < 2 (as the maximal possible value for $\;m_{f}\;$in the *L* = 1 state is 2). If the sample is in the *L* = 1 state it may spontaneously emit light (at 795 nm) reversing the D1 transition (but not necessarily the change in $m_{f}$ as the emitted light is equally likely to emit light with$\;m_{f}=0,1,-1$ ). The result is that atoms begin to accumulate in the *L* = 0, *F *= 2, $m_{f}=2$ state. At this point (as there is no $m_{f}$ =3 state in *L* = 1) the laser light can no longer drive a D1 transition and passes through the vapour without attenuation. This process is schematised over time in (b) where initially the probability of an atom in the *L* = 1 state (due to D1 transition) or the *L* = 0, $m_{f}$ =2 state is low. The action of the laser initially increases the probability of the *L* = 1 state being occupied (due to D1 transitions) but also increases the probability of the *L* = 0, $m_{f}$ =2 state occurring due to optical pumping. As atoms become trapped in the *L* = 0, $m_{f}$ =2 state the probability of D1 transitions drops towards 0 thus rendering the vapour transparent (Figure b is only intended for illustrative purposes and is not intended to be realistic. It was simulated by measuring the frequency of atoms (N = 10000) in a given state following the application of circularly polarised photons to atoms uniformly distributed throughout the ground state Zeeman sub-levels. On some iterations the atoms were allowed to spontaneously emit light with equal probability of $m_{f}=0,\;1,\;-1.\;\;$Note this does not include effects of spin exchange which are to be covered later).

Finally, the hyperfine structure described in the previous section is further split into distinct energy levels in the presence of a magnetic field. This is Zeeman splitting (Zeeman, 1897). As the direction of the laser beam is fixed, we can restrict ourselves to discussing the effects the laser has on the total angular momentum ($\vec{F}$) to a single axis. The description of Zeeman splitting then becomes greatly simplified. Mathematically speaking, we do this by introducing a dimensionless number $\;m_{f},$ which we then multiply by Planck's constant(ℏ) to convert to represent the component of the angular momentum along the laser beam axis ($F_{laser}=\hbar m_{f}$). The allowable values of $m_{f}$ are integral steps between −F and F. This leads to the complete energy level diagram (Fig. 3) for the ground state and first excited state of ^87^Rb. It should be noted that for both *F* = 1 states, negative values of $m_{f}$ give higher energy levels. This is because the different hyperfine states are associated with gyromagnetic ratios of opposite signs (Benumof, 1965).

Now that we have the complete energy level diagram we can describe how shining a laser through the sample results in optical pumping. There are two factors to consider: the effects of the laser and the effect of the atom spontaneously emitting energy. The rules which govern these effects are known as “selection rules”. The effect of the laser is twofold. The first effect is to cause a D1 (ΔL=1) transition while the second effect is to cause the value of $m_{f\;}$ (the component of the angular momentum along the laser axis) to increase ($\Delta m_{f\;}$= 1). For atoms with a single outer shell electron, such as ^87^Rb, this component always increases when the laser light is positively circularly polarised. The polarisation of light is a manifestation of the photon's angular momentum projected on the quantisation axis and thus when we talk about the laser transferring polarisation to the vapour, we really mean that it is transferring angular momentum. Note that if the light was linearly polarised there would not be an accumulation of angular momentum along the axis of the laser (no change in$\;m_{f}$) but D1 transitions would still occur.

The second effect to consider is spontaneous emission (the atom transitioning from the excited, *L* = 1 state to its ground, L=0 state by emitting a photon). Interestingly, and importantly, while the effect on L is reversed the effect on $m_{f}$ is not entirely reversed by spontaneous emission. This is because spontaneously emitted light is equally likely to produce changes of ${\text{m}}_{\text{f}}=0,\;-1,\;1\;.$ In other words, approximately 1/3 of the atoms (that spontaneously emit light) retain their polarisation, while approximately 1/3 lose an increment of polarisation and approximately 1/3 gain the same increment of ${\text{m}}_{\text{f}}$. Therefore, there is a net effect of the optical pumping on the value of ${\text{m}}_{\text{f}}$ whereby it is ‘pumped’ into its highest Zeeman sub level (${\text{m}}_{\text{f}}=2$) of the L=0 state.

Once an atom reaches this state it becomes trapped there. This is because if the laser transfers energy to the vapour it *has to* increase the value of the total atomic angular momentum along the laser axis$\left(m_{f}\right)$. However, there is no energy level with $m_{f}>2$ in the ground or first excited state. Therefore, if the atom already exists in the $L=0,\;F=2,\;m_{f}=2$ state, the light transfers no energy to the sample and simply passes through the vapour unaffected. Effectively, the vapour has become transparent to the laser light. This is optical pumping and is described graphically in Fig. 3.

Once the laser optically pumps the atoms into this transparent steady state, the vapour becomes highly polarised (from here on when we refer to polarisation, this is the polarisation of the gas and not of the laser). This is because many atoms occupy the same state and hence collectively produce a strong net magnetisation (magnetic moment per unit volume, see Eq (3) for relationship between momentum and magnetic moments) which is aligned along the axis of the laser beam. The induced polarisation is highly sensitive to the ambient magnetic field, with any component of the field that is perpendicular to the laser beam producing a torque on the net magnetisation.

Unfortunately, this polarisation is eliminated by relaxation: a process which counters the effects of the laser and returns the vapour to its initial state of poor magnetic sensitivity. Relaxation is a fundamental and unavoidable process but can be minimised by working with rapidly colliding atoms (high density alkali vapours) at zero magnetic field (Happer and Tam, 1977).

### 3.5 Zero field and suppression of relaxation due to spin exchange

If the polarisation, and therefore magnetically sensitive state of the vapour, is to be maintained the precessing atoms must remain in phase. A number of physical processes can cause this phase difference: diffusion of atoms, spin exchange collisions, electron spin randomisation, collisions with the wall of the vapour cell and magnetic field inhomogeneities (Happer, 1972). All these effects can be considered under the umbrella term of relaxation. However in high density alkali vapours spin exchange relaxation is the dominant form of relaxation (Purcell and Field, 1956).

Some optically pumped magnetometers are operated as zero-field magnetometers or SERF (Spin Exchange Relaxation Free) Magnetometers (Allred et al., 2002). This is because in low magnetic fields (<10 nT) relaxation due to spin exchange collisions (the dominant relaxation mechanism) can be supressed greatly, paradoxically by increasing the rate of spin exchange collisions. The two major factors that influence this form of relaxation are magnetic field strength and rate of occurrence of atomic collisions.

When the atoms collide they may exchange electron spin. For instance if atom A (with electron spin = ½) collides with atom B (with electron spin = -½) the sign of their spin may swap. This means that the atoms move between hyperfine energy levels (*F* = 1 - > *F* = 2 and *F* = 2- > *F* = 1). This causes a change in the sign of the gyromagnetic ratio (γ), such that $\gamma \approx +/-\;7Hz\;nT^{-1}\;$(Benumof, 1965). This is known as a spin exchange collision and the rate at which these collisions occur is the spin-exchange rate (R).

In the presence of a magnetic field (B), the individual magnetic moments of the atoms will precess around the field at the Larmor frequency (ω=γB). To maximise sensitivity to external field changes it is key that the atoms precess in phase (this maximises polarisation of the gas). However, if a change in sign of gyromagnetic ratio occurs due to a spin exchange collision, the atoms begin to precess out of phase with each other, reducing the polarisation of the vapour. Considering that we are dealing with many atoms per unit volume ($1.5\;\times \;10^{14}\;cm^{-3}$), a statistical treatment (Happer and Tam, 1977) can be applied to work out the average precessional frequency ($\bar{\omega }$),

(10) $$\begin{array}{c} \bar{\omega }=\;\frac{6I+3}{4I^{2}+4I+3}\gamma B, \end{array}$$

Which is a function of the magnetic field (B) gyromagnetic ratio (γ) and nuclear spin (I). The reason that there is a tendency towards a non-zero average frequency ($\bar{\omega }$) is because there is a greater probability (Happer and Tam, 1977) of the spin system being in the higher energy level (*L* = 0, *F* = 2) as opposed to the lower energy (*L* = 0, *F* = 1) state in the spin exchange regime (ω≪R). The probabilities are governed by the so called “spin-temperature” distribution which says that for a given polarisation (even very small polarisations) there is a tendency for higher energy levels to be preferentially occupied (Anderson et al., 1959; Seltzer, 2008). Perhaps most importantly, the uncertainty (σ) in this mean precessional frequency decreases as a function of spin exchange rate (R),

(11) $$\begin{array}{c} \sigma \propto \;\frac{\gamma^{2}B^{2}}{R}\; \end{array}$$

By decreasing this uncertainty, the atoms are more likely to precess at a frequency close to $\bar{\omega }$ and therefore remain in phase, resulting in a large observable polarisation being maintained for longer (maintaining the magnetic sensitivity of the vapour). If the uncertainty in this system is high (due to a low spin exchange rate: R∼ω), then the spectrum of the precessional frequencies will not cluster around $\bar{\omega }$ and be much more variable, resulting in low polarisation as the atoms are not in phase with one another.

The two ways of achieving this low level of uncertainty involve: (i) decreasing the field (B); (ii) increasing the spin exchange rate (R). The field can reduced by using on board coils (Osborne et al., 2018), external shielding (Holmes et al., 2018; Iivanainen et al., 2019) and by operating the sensors in a mu-metal shielded room. The spin exchange rate can be increased by increasing the density (number of atoms per unit volume) of the rubidium atoms in the sensor (making collisions more likely to occur). The density is increased by heating the cell (increasing the vapour pressure), increasing the number density of the atoms in the vapour phase. This temperature can be quite high; in the case of ^87^Rb it is necessary to heat the cell to ∼150° to achieve sufficient vapour density (Shah et al., 2007). Practically, this means the cell should be offset from the sensor walls (increasing distance to the brain) so the temperature of the sensor exterior does not exceed ∼40°. Importantly, if a different vapour is used, such as ^4^He, heating is not required, but at the cost of reduced sensitivity (Labyt et al., 2018).

The take home message is that when the rate of spin exchange collisions (due to high atomic density) greatly exceeds the precessional frequency (which is low due to operating at very low field), a strong polarisation can be maintained in the vapour.

## 4 Signal equations

### 4.1 Magnetic fields and matter: a steady state solution

In the SERF regime relaxation is suppressed and, as we have seen a tuned laser can pump most of the atoms into the same energy level, a large steady state polarisation (alignment of magnetic moment per unit volume) within the vapour is created along the axis of the laser. The behaviour of this polarisation is highly sensitive to magnetic fields and can be described phenomenologically using the Bloch Equations (Bloch, 1946). The rate of change of polarisation of the vapour is equal to the cross product of the atomic polarisation ($\vec{P}$) with the magnetic field ($\vec{B}$) times the gyromagnetic ratio (γ).

(12) $$\begin{array}{c} \frac{d\vec{P}}{dt}=\;\gamma \vec{P}\;\times \vec{B} \end{array}$$

This cross product can be expanded in terms of its vector components,

(13) $$\begin{array}{c} \frac{dP_{x}}{dt}=\;\gamma \left(P_{y}B_{z}-\;P_{z}B_{y}\right), \end{array}$$

(14) $$\begin{array}{c} \frac{dP_{y}}{dt}=\;\gamma \left(P_{z}B_{x}-\;P_{x}B_{z}\right), \end{array}$$

(15) $$\begin{array}{c} \frac{dP_{z}}{dt}=\;\gamma \left(P_{x}B_{y}-\;P_{y}B_{x}\right), \end{array}$$

The above equations determine how the polarisation of the vapour changes due to the external magnetic field. However, the vapour is also being simultaneously optically pumped, re-establishing the equilibrium polarisation, $P_{0}$ (governed by the time constant $\;\;T_{p}$ – a recovery effect) and relaxing back to its ground state (governed by time constant T-a decay effect). If the optical pumping takes place along $P_{x}\;$ then the differential equations update as follows to account for the pumping ($\;T_{p}$) and simultaneous relaxation (T),

(16) $$\begin{array}{c} \frac{dP_{x}}{dt}=\;\gamma \left(P_{y}B_{z}-\;P_{z}B_{y}\right)+\frac{P_{0}-P_{x}}{T_{p}}-\frac{P_{x}}{T}, \end{array}$$

(17) $$\begin{array}{c} \frac{dP_{y}}{dt}=\;\gamma \left(P_{z}B_{x}-\;P_{x}B_{z}\right)-\frac{P_{y}}{T}-\frac{P_{y}}{T_{p}}, \end{array}$$

(18) $$\begin{array}{c} \frac{dP_{z}}{dt}=\;\gamma \left(P_{x}B_{y}-\;P_{y}B_{x}\right)-\frac{P_{z}}{T}-\frac{P_{z}}{T_{p}} \end{array}$$

During sensor operation the ambient fields are nulled using on-sensor or external coils. Here we assume, for simplicity, that *B*_*x*_* *= *B*_*y*_ = 0 and focus on the effect of small changes in *B*_*z*_ on the polarisation. These are characterised by:

(19) $$\begin{array}{c} \frac{dP_{x}}{dt}=\;\gamma P_{y}B_{z}+\frac{P_{0}-P_{x}}{T_{p}}-\frac{P_{x}}{T}, \end{array}$$

(20) $$\begin{array}{c} \frac{dP_{y}}{dt}=\;-\gamma P_{x}B_{z}\;-\frac{P_{y}}{T}-\frac{P_{y}}{T_{p}}, \end{array}$$

(21) $$\begin{array}{c} \frac{dP_{z}}{dt}=\;\;-\frac{P_{z}}{T}-\frac{P_{z}}{T_{p}} \end{array}$$

The following substitutions are often made to simplify the calculations:

(22) $$\begin{array}{c} \frac{1}{\tau }=\;\frac{1}{T_{p}}+\frac{1}{T}, \end{array}$$

(23) $$\begin{array}{c} P_{0}^{'}=\;P_{0}\frac{T}{T_{p}+T} \end{array}$$

Eq (22) combines the time constants T and $T_{p}$ in to a single effective relaxation time τ, while Eq (23) rescales the equilibrium polarisation to reflect the effects of relaxation. Intuitively the second term simply states that if the effect of the natural relaxation rate $\;\;\left(\frac{1}{T}\right)$ is large relative to the optical pumping rate $\left(\frac{1}{T_{p}}\right)$ then the effective polarisation $\left(P_{0}^{'}\right)$ will be significantly less than the equilibrium polarisation. This should not be the case because this form of relaxation is reduced by operating in the spin exchange relaxation free regime (high density, low field, (Ledbetter et al., 2008)). Using these substitutions the differential equations can be recast as:

(24) $$\begin{array}{c} \frac{dP_{x}}{dt}=\;\gamma P_{y}B_{z}+\frac{P_{0}^{'}-P_{x}}{\tau }, \end{array}$$

(25) $$\begin{array}{c} \frac{dP_{y}}{dt}=\;-\gamma P_{x}B_{z}\;-\frac{P_{y}}{\tau }, \end{array}$$

(26) $$\begin{array}{c} \frac{dP_{z}}{dt}=\;\;-\frac{P_{z}}{\tau } \end{array}$$

We can now find the steady state solution to these equations which describes the combined effects of the optical pumping and magnetic field on the polarisation. This leads to the following set of equations.

(27) $$\begin{array}{c} P_{x}=\;\frac{P_{0}^{'}}{1+{\left(\gamma B_{z}\tau \right)}^{2}}, \end{array}$$

(28) $$\begin{array}{c} P_{y}=\;\frac{-\gamma B_{z}\tau \;P_{0}^{'}}{1+{\left(\gamma B_{z}\tau \right)}^{2}}, \end{array}$$

(29) $$\begin{array}{c} P_{z}=\;0 \end{array}$$

**Fig. 4:**
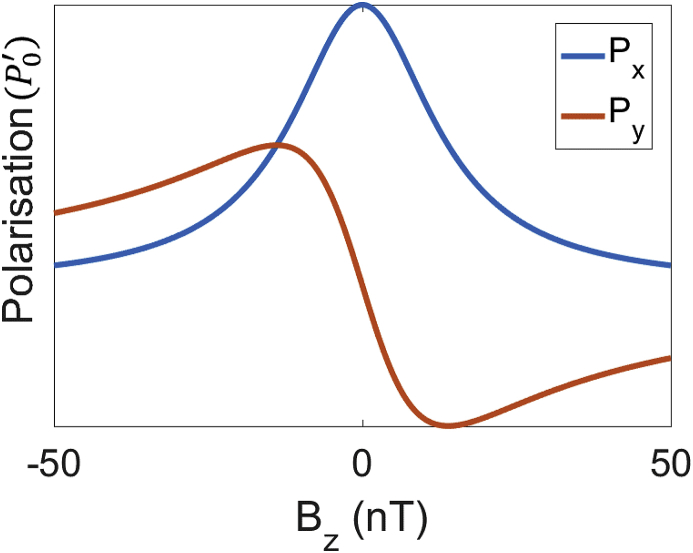
The polarisation along the axis of the laser $\;\left(P_{x}\right)$ displays an absorption profile while the polarisation along the other axis $\;\left(P_{y}\right)$ has a dispersion shape. In both cases the presence of magnetic field causes a polarisation change that is a function of the effective equilibrium polarisation ($P_{0}^{'}$).

The curves defined by these equations are known as absorption ($P_{x}$) and dispersion curves ($P_{y}$) in the spectroscopy literature (Fig. 4).

While these curves do show a strong sensitivity to small magnetic field changes, such measurements can be quite sensitive to low-frequency 1/f noise (Osborne et al., 2018). The solution to this issue is to produce an amplitude modulation of the polarisation and to measure this modulation using lock-in detection (Cohen-Tannoudji et al., 1970; Kastler, 1973). However, if we are to account for the effects of amplitude modulation in the signal equation a simple steady state algebraic solution is no longer valid.

### 4.2 Magnetic fields and matter: in the presence of amplitude modulation

The amplitude modulation utilised by zero field OPM sensors often involves using on board coils to generate an oscillating magnetic field that varies at a frequency much greater than the relaxation rate of the vapour so that polarisation is amplitude-modulated at a frequency outside the bandwidth of the sensor (Cohen-Tannoudji et al., 1970). The relaxation rate of the vapour is ∼100 Hz (the equivalence between relaxation rates and bandwidth is explored later) while the modulating field has a frequency of typically 1000 Hz. Before introducing this effect to the signal equation we first make a substitution utilising complex notation for the transverse polarisation ($P_{xy}$): $P_{xy}=P_{x}+iP_{y}.$ This give the Bloch equations a slightly different form that allows simultaneous solutions for $\;P_{x}$ and $P_{y}$ to be derived. This gives

(30) $$\begin{array}{c} \frac{dP_{xy}}{dt}=\;\frac{P_{0}^{'}-P_{xy}}{\tau }+i\gamma \left(P_{xy}B_{z}\right), \end{array}$$

(31) $$\begin{array}{c} \frac{dP_{z}}{dt}=\;\;-\frac{P_{z}}{\tau }. \end{array}$$

With the addition of an oscillating magnetic field of amplitude $\;B_{1}$and angular frequency ω applied along the z-axis Eq. (30) becomes.

(32) $$\begin{array}{c} \frac{dP_{xy}}{dt}=\;\frac{P_{0}^{'}-P_{xy}}{\tau }+i\gamma P_{xy}\left(B_{z}+B_{1}\;\cos\;\omega t\right), \end{array}$$

while Eq (31) is unaltered. With this formulation it is not possible to use the simple steady state (time independent) solution as $\frac{dP_{xy}}{dt}$ changes over time due to the presence of the cosine term. However, a Fourier series solution can be formed. The dominant term in the solution is the first harmonic which is described by Cohen-Tannoudji et al. (1970):

(33) $$\begin{array}{c} P_{x}\left(t\right)=\;P_{0}^{'}\;J_{0}\left(\frac{\gamma B_{1}}{\omega }\right)J_{1}\left(\frac{\gamma B_{1}}{\omega }\right)\;\frac{\gamma B_{z}\tau }{1+{\left(\gamma B_{z}\tau \right)}^{2}}\sin\;\omega t \end{array}.$$

Here, $J_{n}$ are Bessel functions of the first kind. While the inclusion of the Bessel functions complicate the interpretation of the signal equation they evaluate to a constant term and thus can simply be considered a s a constant of proportionality ($A_{0}$) which absorbs the P0' term. As alluded to in earlier sections, $P_{x}\left(t\right)\;$ is not measured directly but instead the intensity/polarity of the laser (or probe laser in the pump/probe setup) is measured using a photodiode/polarimeter (voltage). The voltage (V) measured by the detector is demodulated with a lock in amplifier and the sine term is removed from the signal equation which now has the following form

(34) $$\begin{array}{c} V\left(B_{z}\right)=\;\;A_{0}\;\frac{\gamma B_{z}\tau }{1+{\left(\gamma B_{z}\tau \right)}^{2}} \end{array}$$

Due to the amplitude-modulation, the SNR of the system improves (Kastler, 1973) having shifted the signal away from the low frequency end of the spectrum (Osborne et al., 2018). Furthermore, the relationship between voltage and magnetic field now no longer follows an absorption curve (as it does in the case without amplitude modulation), but instead is now a dispersion curve which means that positive and negative field changes can be discriminated (Fig. 4).

### 4.3 Linearity of response

As described in the previous section the response of the system with respect to changes in the magnetic field is non-linear. Specifically the system response is that of a dispersion curve. However, this non linearity is only noticeable when one looks across a wide range of magnetic fields. In the zero field regime, where $\;\gamma B_{z}\tau \ll 1$, the response is, in fact, linear and this can be verified by examining the Taylor series of the signal equation around $\;B_{z}=0$. The linear term in the Taylor series for the signal equation has the following form

(35) $$\begin{array}{c} V\left(B\right)=A_{0}\;\gamma B_{z}\tau \end{array}$$

**Fig. 5:**
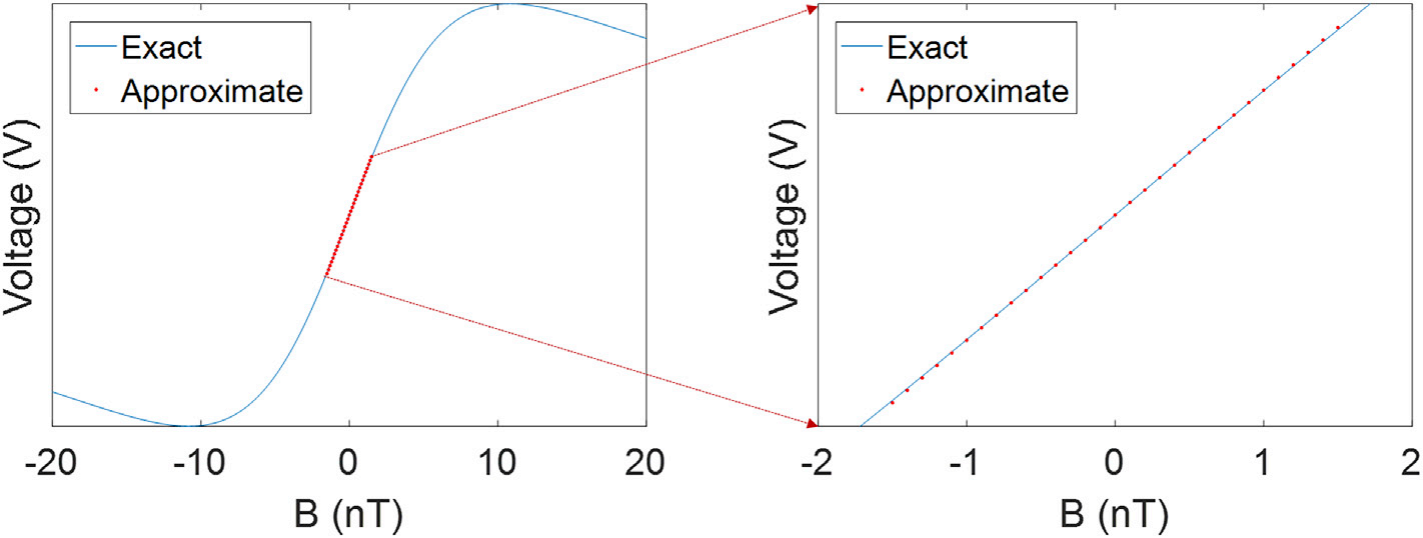
In the left hand panel the response of the system is plotted over a 40 nT range. In this range the response of the system is clearly non-linear. However if one examines the curve within the dynamic range of the sensors ( ±1.5 nT) a linear approximation to the curve produces a less than 1% deviation at 1 nT (right hand panel). It should be noted that this deviation will differ between different sensor designs and assumes that the transverse fields are close to zero.

This is simply the equation of a straight line and its fit to the exact solution can be seen in Fig. 5. It should be noted that this form assumes that the components of the magnetic field ($B_{x}\;$ and$\;B_{y}$) are close to zero. If not, this may result in calibration errors that should be corrected with an active shielding approach (Iivanainen et al., 2019).

A number of things should be clear from the above representation. First, the relationship between the observed signal and the magnetic field is only linear within finite range of values. This means that when a sensor experiences a large magnetic field a non-linearity will be introduced into the response. Second, now that the equation is in a linear form, sensitivity can intuitively be related to the slope of this graph: a steep slope indicates that a large change in signal (voltage) is observed for a small change in magnetic field. Therefore, if we want to change sensitivity we need to alter the slope of this graph. Importantly there is a trade-off here between non-linearity and sensitivity. If the sensitivity of the magnetometer is reduced, the slope of the response decreases but is linear over a wider range of field values. The compromise between sensitivity and linearity/dynamic range is therefore application specific.

### 4.4 Improving or compromising sensitivity

If we wish to alter sensitivity there are two variables in the linear signal (Eq (35)) of interest equation: γ &τ – the gyromagnetic ratio and the relaxation time respectively. The gyromagnetic ratio is fixed for a given atom and cannot be changed so therefore the relaxation time is the predominant way to change sensitivity. By increasing the relaxation time (τ) this curve becomes steeper and thus the sensitivity is increased. This is most easily achieved by increasing the temperature which increases rate of spin exchange collisions (*R*) and therefore maintains larger polarisation (Eq (11)) for longer (increases τ). However, there is a fundamental drawback to this approach. Once the relaxation time (τ) increases, the bandwidth of the system drops. This can be seen more easily when one considers that the magnitude spectrum has the following form (Shah and Wakai, 2013) as a function of relaxation time (τ),

(36) $$\begin{array}{c} M\;\left(f,\tau \right)=\;\frac{1}{\sqrt{1+{\left(f\tau \right)}^{2}}}, \end{array}$$

where Mis the magnitude, f is the frequency of field variation in Hz andτ is the relaxation time (seconds). If one chooses to define the 3 dB point as the bandwidth (BW) of the system a simple relationship between BW and τ is observed by letting $M\;\left(f,\tau \right)=\;\frac{1}{\sqrt{2}}$ and expressing in terms of f.

(37) $$\begin{array}{c} f=\frac{1}{\tau }=BW \end{array}$$

This is because the relaxation time is a time constant that determines how long we must wait before another independent measurement can be made. As such there is a fundamental physical tension between altering sensitivity and achieving the most desirable bandwidth. This is more precisely expressed in the following equation (Allred et al., 2002) quantifying the smallest measurable change in field (ΔB).

(38) $$\begin{array}{c} \Delta B=\;\frac{1}{\gamma \sqrt{n\tau Vt}}, \end{array}$$

where n is equal to the density of the atoms, τ is the relaxation time, V is the volume of the cell, γ is the gyromagnetic ratio and t is the integration time. If the integration time (t) is chosen to be half the relaxation time (equivalent to a sampling frequency of twice the bandwidth) we get the following expression for sensitivity expressed in terms of bandwidth (BW) when we express the relaxation time (τ) also in terms of bandwidth ($\frac{1}{\tau }=BW$).

(39) $$\begin{array}{c} \Delta B=\;\frac{\sqrt{2}\;BW}{\gamma \sqrt{nV}} \end{array}$$

In other words, sensitivity can be increased by increasing atomic density or cell volume, but decreases with increasing measurement bandwidth. As an example, for ^87^Rb with $\;\gamma =7Hz\;nT^{-1}$, n  =  $1.5\;\times \;10^{14}\;cm^{-3}$ and $V=0.027cm^{3}$ and BW=100Hz results in a theoretical sensitivity of 10fT/√Hz.

### 4.5 A bound on cross-talk induced gain changes

We have aimed so far to provide an intuitive linear approximation for the signal equation for OPMs. However to make this approximation we have assumed that the Bessel functions in Eq (33) evaluate to a constant term. This is true for a single channel OPM system but is not necessarily true for a multi-channel OPM system. This is because any magnetic field produced by a sensor, whether to modulate polarisation or to zero the DC magnetic field can affect a nearby sensor. This is a non-trivial problem as the amplitude of the modulating field may be 50–100 nT and the amplitude of the DC zeroing fields may be in the range of 2–50 nT. Luckily, the cross-talk due to the DC zeroing fields can be minimised by using external field nulling coils (Holmes et al., 2018) which can reduce the magnitude of the DC field to hundreds of picotestla. Therefore in this regime the cross-talk is dominated by the action of the modulating fields. We will consider the effects of this modulating field in much greater detail now.

In a previous section we described how amplitude modulation of the polarisations alters the fundamental signal equation. This modulation allows for detection of signal along multiple axes and also reduces noise in the system. If one sensor is operated in isolation the effective amplitude of the modulation field is unchanged. However, if two sensors are operated in close proximity the modulation coil of one sensor may change the amplitude of the modulation field on the other sensor. This is because the modulation is itself a magnetic field. In the case where the modulation fields of all sensors are in phase their modulation fields constructively interfere and create a new modulation amplitude that is different from the optimal amplitude at each sensor. Here we present a theoretical analysis of how the negative effects of this cross-talk manifest and how the linear approximation to the signal equation should be modified in order to model cross-talk. The signal equation, as previously stated (Eq (33)), contains terms characterising the modulation field amplitude $\;\left(B_{1}\right)$ and frequency (ω) (Cohen-Tannoudji et al., 1970). If $B_{1}$ increases or decreases a change in gain (G) occurs which can be modelled as a ratio between the signal equation without cross-talk and in the presence of cross-talk (assuming $\;B_{1}\;$ is parallel to $\;B_{2}$).

(40) $$\begin{array}{c} G=\;\frac{P_{0}^{'}\;J_{0}\left(\frac{\gamma B_{2}}{\omega }\right)J_{1}\left(\frac{\gamma B_{2}}{\omega }\right)\;\frac{\gamma B_{z}\tau }{1+{\left(\gamma B_{z}\tau \right)}^{2}}\sin\;\omega t}{\;P_{0}^{'}\;J_{0}\left(\frac{\gamma B_{1}}{\omega }\right)J_{1}\left(\frac{\gamma B_{1}}{\omega }\right)\;\frac{\gamma B_{z}\tau }{1+{\left(\gamma B_{z}\tau \right)}^{2}}\sin\;\omega t}=\frac{J_{0}\left(\frac{\gamma B_{2}}{\omega }\right)J_{1}\left(\frac{\gamma B_{2}}{\omega }\right)}{J_{0}\left(\frac{\gamma B_{1}}{\omega }\right)J_{1}\left(\frac{\gamma B_{1}}{\omega }\right)}, \end{array}$$

where $B_{2}$ is the amplitude of the modulation field in the presence of cross-talk. We can simplify this product by utilising the asymptotic form for the Bessel function (assuming $\;0<x<\sqrt{\alpha +1}$) of order α. This condition will be satisfied when ${\;\text{γB}}_{1}$ <ω which is often the case as making ${\text{B}}_{1}$ large relative to ω runs the risk of increasing relaxation and reducing the sensitivity of the sensor (Shah and Romalis, 2009).

(41) $$\begin{array}{c} J_{\alpha }\left(x\right)∼\frac{1}{\text{Γ}\left(\alpha +1\right)}{\left(\frac{x}{2}\right)}^{\alpha }, \end{array}$$

where Γ is the gamma function. The product of the Bessel functions then reduces to a very simple form.

(42) $$\begin{array}{c} J_{0}\left(x\right)J_{1}\left(x\right)=\;\;\frac{x}{2} \end{array}$$

The gain change has an even simpler form.

(43) $$\begin{array}{c} G=\;\frac{J_{0}\left(\frac{\gamma B_{2}}{\omega }\right)J_{1}\left(\frac{\gamma B_{2}}{\omega }\right)}{J_{0}\left(\frac{\gamma B_{1}}{\omega }\right)J_{1}\left(\frac{\gamma B_{1}}{\omega }\right)}=\frac{B_{2}}{B_{1}} \end{array}$$

If we define $B_{2}$ to be the modulating field amplitude $B_{1}$ plus some error field $B_{\varepsilon }$ then we can reframe the gain change in terms of cross-talk.

(44) $$\begin{array}{c} G=\frac{B_{2}}{B_{1}}=\frac{B_{1}+B_{\varepsilon }}{B_{1}}=1+\frac{B_{\varepsilon }}{B_{1}} \end{array}$$

Considering that the cross-talk (CT) is the ratio of this error field to the amplitude of the modulating field the gain can be simply stated as follows,

(45) $$\begin{array}{c} G\;=\;1+CT \end{array}$$

**Fig. 6:**
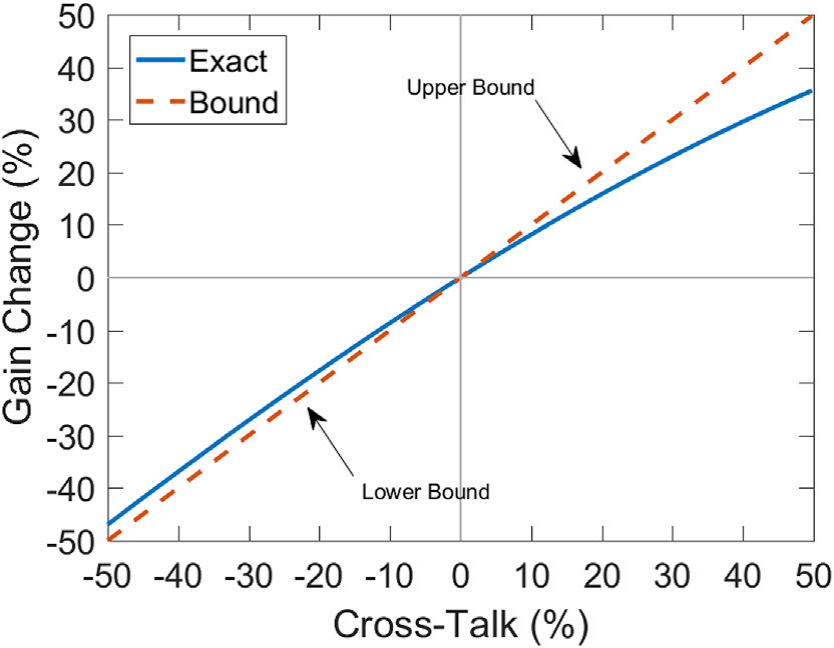
Bounding the relationship between cross-talk and gain. The exact solution (eq (43)) is derived using the normalised product of a zeroth and first order Bessel function when $\;B_{2}$ and $B_{1}$ are parallel. The gyromagnetic ratio is assumed to be 7 Hz nT^−1^, the true amplitude of the modulating field is assumed to be 60 nT and the frequency of the field is 923Hz (based on QuSpin OPMs, but the bound is still valid for any OPM utilising modulating fields as long as $\frac{\gamma B_{1}}{\omega }<1.08$-maximum of the Bessel function product). Left of the origin the approximation is a lower bound on the cross talk induced gain changes while right of the origin it is an upper bound on the cross-talk induced gain changes. Note that these curves deviate further from linearity for positive cross-talk. It should also be noted that for realistic values of cross-talk the ( ±10%) this bound is a reasonable approximation for both positive and negative gain changes.

Interestingly, this formulation does not just approximate the gain changes due to cross-talk but also provides a bound on the gain changes in the situation where $B_{2}$ and $B_{1}$ are parallel. This is a valid bound when the argument of the Bessel function in the absence of cross-talk is less than the function maximum ($\frac{\gamma B_{1}}{\omega }<1.08$). This can be verified by inspection in Fig. 6.

### 4.6 The final signal equation

Fig. 6 shows that the predicted gain changes due to cross-talk will be bounded by the percentage change of the amplitude of the modulation field (e.g. 3% at 20 mm). It is interesting that the curve is asymmetric (the effects of negative cross-talk being closer to linearity); but importantly in the low cross-talk regime (<10%) this bound is a suitable approximation for both positive and negative cross-talk. Note that we are assuming that the additional modulating field does not move the sensor out of the zero field regime. This is typically the case as large amplitude modulating fields can decrease sensor performance (by decreasing the relaxation time constant) and are therefore less frequently employed (Shah and Romalis, 2009). We can now include this effect in the linear approximation to the OPM signal equation. We can also highlight the relationship between signal and bandwidth by expressing the relaxation time in terms of bandwidth $\;\left(\frac{1}{\tau }=BW\right)$.

(46) $$\begin{array}{c} V\left(B_{z},BW,CT,\gamma \right)=\left(A_{0}\right)\left(\gamma \right)\left(BW^{-1}\right)\left(B_{z}\right)\left(1+CT\right)\; \end{array}$$

The signal equation (expressed as a voltage: V) for a multichannel OPM system can now be understood simply in terms of 5 intuitive terms: $A_{0}$ (proportionality constant/calibration factor), γ (vapour dependent property), $B_{z}$ (the magnetic field changes), BW (the bandwidth in Hz) and CT (The fraction of cross-talk). This form for the signal equation is quite informative as it relates important quantities such as bandwidth, magnetic field changes and cross-talk to the observable voltage signal. This formulation also leads to a simple conclusion. The signal equation is linear but the slope of the output voltage w.r.t. applied field will change as a function of cross-talk. Although this effect is small (∼3%), practically, for sensors with shared modulation frequency and phase, this would mean that any changes in array geometry should ideally be followed by a re-calibration.

## 5 Discussion

In summary this article provides a theoretical overview of the physics surrounding optically pumped magnetometers. We hope that this introduction will serve as a useful stepping stone between the developing neuroscientific literature and the extant physics literature on these devices.

The key points to take away from this article are as follows.

1. The simple atomic structure of alkali metal vapours allows for optical pumping
2. A laser of the correct frequency will drive a D1 (or D2) transition.
3. If the laser is circularly polarised, this D1 transition will be accompanied by an increment in angular momentum along the laser axis
4. Eventually the atoms in the vapour become trapped in one energy state (no longer absorbing laser light) creating a large polarisation in the vapour.
5. At low field and at high atomic density this polarisation can be maintained for longer.
6. Detection via modulation of this polarisation with on board coils improves signal to noise
7. At low field this modulated polarisation varies linearly with respect to magnetic field
8. The greater the volume of vapour the higher the sensitivity
9. There is a tradeoff between measurement bandwidth and sensitivity
10. The percentage cross-talk between sensors provides bounds on the cross-talk induced gain changes.

In this review we have focused on small independent devices with the potential to form multi-channel arrays operating at close to zero field. Depending on the eventual purpose of the device many optimizations can clearly be made. For instance it has been shown that OPMs can have less than a 1fT/√Hz intrinsic white noise level (Allred et al., 2002; Kominis et al., 2003; Lee et al., 2006) yet most OPMs used for MEG, to date, have operated with sensitivities on the order of 10–100 fT/√Hz (with slight variations in sensitivity throughout the typical 1–100Hz bandwidth utilised for MEG).

Rather than directly aiming to improve the white noise level of the sensors a number of groups have aimed to improve interference rejection by constructing atomic gradiometers. An optimal method of doing this has yet to emerge and multiple approaches exist. For instance one approach involves the use of a diffractive element to split the laser beam into multiple components, passing through the same vapour cell, forming four separate channels, from which gradiometers can be constructed (Colombo et al., 2016). Others utilise multiple lasers (Fang et al., 2014) or multiple vapour cells in order to achieve a gradiometric configuration ( Sheng et al., 2017a). All these approaches offer the possibility to reduce magnetic interference and noise from the laser which ultimately should improve the SNR of a given experiment. This would be particularly exciting for MEG as dealing with the magnetic interference observed by OPMs can be challenging. For instance, the drifting of the environmental magnetic field over time will cause a gain change in the sensor due to the subtle departure from the zero field regime (Iivanainen et al., 2019). Some sensors operate in a closed loop setting in order to dynamically track this zero (Labyt et al., 2018) but this is atypical for the majority of OPMs currently in use for MEG.

One important factor that will have to be addressed in future system designs and modelling studies is the effect of cross-talk due to amplitude modulation of the vapour polarisation. We have given a very simplistic overview that aims to provide an intuitive account for how cross talk affects an OPM system. We note that the theoretical account we present has several limitations. Most notably, the model assumes that the product of the gyromagnetic ratio and amplitude of the modulation field is small relative to the frequency of the modulation field. This may not be the case for all sensor designs as there are trade-offs to consider between the amplitude of the signal and the extra relaxation induced by the modulation field (Shah and Romalis, 2009). Secondly, we have assumed that the interfering modulation field (experienced by the other cell) is homogeneous across the cell volume. The will not be the case for local, high-gradient fields and will effectively increase the relaxation rate of the vapour, lowering sensitivity. Finally, the interfering modulation field will perturb sensitive axis of the sensor, this will give rise to forward modelling errors may affect the quality of source estimation (Zetter et al., 2017).

However, on an empirical note we have previously observed maximum cross-talk between sensors to be on the order of 3% (Boto et al., 2018) at distances of ∼20 mm. Although small, this clearly needs to be incorporated into our models and will be the subject of further study (Roberts et al., 2018). This is an interesting consideration of sensor design that is unique to multi-channel systems. We also have only focused on between sensor cross-talk. We have neglected the interactions between the radial and tangential measurements within the sensor. Typically, MEG experiments measure fields that are radial to the head, but many systems can measure fields that are both radial and tangential to the head simultaneously with the addition of an extra modulation field (Borna et al., 2017b; Osborne et al., 2018). It is possible that these fields may interfere with each other and cause gain changes. This should be a direction of future study so that the neuroscience community can take full advantage of the extra information offered by these measurements (Iivanainen, Stenroos and Parkkonen, 2017b).

Throughout this review we have not discussed the practical constraints on manufacturing and cost that working with OPMs incurs. With this in mind many OPM systems are currently constrained by many of the same costs as SQUID systems requiring a heavily (and costly) magnetically shielded room in which to operate. They also require additional field nulling-coils within the room if the subject is to be allowed to move (Holmes et al., 2018). However, there are OPM devices which can operate within the Earth's field (dispensing with the need for costly shielding), but, have until recently, been subject to much higher noise levels (Seltzer and Romalis, 2004). A recent study demonstrated a pulsed optically pumped magnetometer that can operate in unshielded environments with a sensitivity rivalling that of the zero field magnetometers (Cooper et al., 2016). This represents an incredibly exciting advancement as the operation of OPMs within ambient fields could lead to a rapid increase in the application of these devices in clinical neuroscience.

For those who are now considering engaging in neuroscientific research with OPMs there are a number of factors that should be considered beforehand. Firstly, as mentioned earlier they still necessitate a magnetically shielded room. Secondly, the shielded room should have homogenous and small static magnetic fields (<1 nT). If not, active shielding (Holmes et al., 2018) will be required to further enhance the homogeneity of the room. This will be less of an issue if the experimental question does not require subject movement. In that case the OPM array can be made static (similar to cryogenic MEG systems). The environment outside the shielded room is also quite important. For instance a shielded room in a busy city will pick up substantial low frequency interference from the environment (In London the shielded room is directly above an underground train line). This problem will be site specific and require real time active shielding to compensate for the interference (Iivanainen et al., 2019). One must also consider whether the bandwidth, dynamic range and sensitivity of the OPM is sufficient to answer the specific neuroscientific question of interest. For all these reasons installing an OPM system in a neuroscience environment will require close collaboration between physicists and neuroscientists.

Furthermore, OPMs are currently not sold as ready to use MEG systems. An electronics system will need to be set up (analogue to digital converters, current drivers for coils, triggering systems for cognitive experiments). From the software perspective there is no agreed upon file formats for OPM data and no standardised acquisition software (as the system will be custom). We have created a standard binary file format for raw data storage and all metadata is stored in. json and. tsv files. The aim of this approach was to align the data format as much as possible with the BIDS format for MEG (Niso et al., 2018) so that data can be easily read across different software packages. We provide example and test data for use with SPM12 (available at https://github.com/tierneytim/OPM) which we hope might become more widely used. The final practical issue concerns how sensors are located relative to the brain: the coregistration problem. Solutions to this problem have been to use custom scanner-casts which are subject specific, but offer maximal sensitivity (Tierney et al., 2018) or to use structured light scanners to measure the sensor positions (Zetter et al., 2019) in a quick and inexpensive fashion. Undoubtedly, these practical issues will be resolved with time but the interested neuroscientist should be aware of them before taking the plunge into the world of OPMs.

In conclusion, we have reviewed the theory of OPMs from their quantum origins to their use in multi-channel MEG systems. The multi-disciplinary interaction between physics, engineering and neuroscience has brought OPM technology to a point where it is beginning to transform the experiments we can do and the neuroscientific questions we can ask.

## Conflicts of interest

Co-author Vishal Shah is a director at QuSpin – a commercial entity selling OPMs. The remaining authors are academic scientists who have no commercial interests.
